# Analysis of CT and MR imaging features of the brain in patients with hydrogen sulfide poisoning based on clinical symptom grading

**DOI:** 10.1186/s12883-022-02956-z

**Published:** 2022-11-07

**Authors:** Daidi Tang, Ning Tian, Jianming Cai, Jinlin Ma, Tingting Wang, Hongtao Zhang, Fugeng Sheng

**Affiliations:** Department of Radiology, the Fifth Medical Center of Chinese PLA General Hospital, Dongda Street 8, Beijing, 100071 China

**Keywords:** Hydrogen sulfide poisoning, Brain injury, CT, MRI

## Abstract

**Objective:**

To retrospectively analyze CT and MR imaging features of the brain in patients with hydrogen sulfide poisoning based on clinical symptom grading and to investigate their correlations with clinical symptoms and patients’ prognosis.

**Methods:**

A retrospective analysis was performed of CT and MR imaging data of the brain in 40 patients with hydrogen sulfide poisoning in our hospital. There were four main imaging manifestations. Patients were clinically graded according to the central nervous system symptom scores of the Poisoning Severity Score (PSS) and staged according to the gas inhalation time segment. Based on clinical symptom grading, the frequencies and proportions of four imaging signs that occurred in each group were counted, their development trends were analyzed, and the correlations of imaging features with clinical grading and prognosis were calculated.

**Results:**

Forty patients were divided into minor, moderate and severe clinical grades and classified into four stages. In patients with minor and moderate clinical grading, only one patient suffered from generalized brain edema at stage 1, with a good prognosis. Patients with severe clinical grade showed the highest probability of presenting with the four imaging signs. The imaging signs were correlated with the severe clinical grade and a poor prognosis (*P* = 0.000, R = 0.828; *P* = 0.000, R = 0.858).

**Conclusion:**

In patients with the severe clinical grade, generalized brain edema and symmetrical hypodensity/abnormal signals in the bilateral basal ganglia and around the lateral ventricles were the main findings and were shown to persist. The presence of imaging signs can assist in the clinically effective evaluation of clinical symptom grade.

## Background

Hydrogen sulfide (H_2_S) is a fatal environmental and industrial toxicant that is second only to carbon monoxide poisoning in patients with occupational acute poisoning in China. The gas is virulent, colorless, flammable, and harmful, with a characteristic odor of rotten eggs at low concentrations. Hydrogen sulfide is widely present in low-lying spaces, such as underground pipes, mines, manure pits, and biogas pools. On special occasions, in summer, dead fish in a fish hold may rapidly decompose to release harmful gases such as H_2_S [1, 2]. Additionally, reduced oxygen content can cause acute poisoning, involving the central nervous system, respiratory system, heart, liver, and other organs [3, 4]. Airway inhalation is a main poisoning route. Because the central nervous system is the most sensitive organ system to hypoxia, it will be damaged first, and specially, hypoxic encephalopathy is the most significant [5, 6]. The disease conditions of poisoning patients progress rapidly, with very high mortality. It has been reported in China that the mortality of occupational acute hydrogen sulfide poisoning is 23.1–50% [7, 8]. CT and MRI of the brain are of great importance in diagnosing brain injury induced by hydrogen sulfide poisoning, but this has rarely been reported in the literature. The present study aims to analyze the CT and MR imaging features of the brain in patients with hydrogen sulfide poisoning based on staging according to different gas inhalation times and to investigate correlations of minor, moderate and severe clinical symptoms and patients’ prognoses with the severity of imaging findings to assist in clinical diagnosis and treatment.

## Methods

### Study population

Forty patients (35 males and 5 females; aged 18–54 years; median age, 37.5 years) with hydrogen sulfide poisoning in our hospital were enrolled from January 1, 2013 to December 30, 2018. All patients underwent CT or MRI of the brain successfully. All patients enrolled were previously healthy and complained of no disorders of the heart, liver, lungs, and kidneys. Of the 40 patients who experienced hydrogen sulfide poisoning, 11 acquired the poisoning while working in underground operations, 8 in hydrogen sulfide production workshops, 7 in tanneries, 5 in road and bridge work, 4 in sewage treatment plants, and 1 each while working in a fish tank, septic tank, chemical plant, oil refinery, and paper mill.

### Examination equipment

CT scanner were performed with a GE 64-slice spiral CT device (GE LightSpeed VCT). Patients were placed in the supine position and scanned from the vertex of the skull to the skull base. The tube voltage was 140 KV, the tube current was 335 mA, the slice thickness was 5 mm, the slice interval was 5 mm, and the matrix was 512 × 512, with FOV:320.

For MRI of the brain, a GE Signa HD 1.5 T MRI scanner (GE, USA) or Espree 1.5 T MRI System (SIEMENS, Germany) was used. Using a head coil, patients were placed in the supine position and scanned from the roof of the skull to the skull base. The fixed slice thickness was 5.0 mm, and the slice interval was 1.5 mm. Regarding the scanning sequences, plain scans were obtained with transverse T2WI, T1WI, DWI, sagittal T1-FLAIR-FS, and coronal T2-FLAIR.

### Clinical poisoning grading standards

In the present study, case-related clinical poisoning grading was performed according to the nervous system symptoms in the Poisoning Severity Score (PSS) (proposed by the European Association of Poisons Centers and Clinical Toxicologists (EAPCCT) in 1990 and widely adopted in Europe since 1994):

1. Minor: drowsiness, vertigo, tinnitus, and ataxia; restlessness; mild extrapyramidal symptoms; mild cholinergic or anticholinergic symptoms; paresthesia; and mild visual or auditory disturbances.
2. Moderate: unconsciousness with appropriate response to pain; brief apnea and bradypnea; confusion, agitation, hallucinations, and delirium; infrequent, generalized or local seizures; pronounced cholinergic or anticholinergic symptoms; localized paralysis not affecting vital functions; and visual or auditory disturbances.
3. Severe: deep coma with inappropriate response to pain or unresponsive to pain; respiratory depression with insufficiency; extreme agitation; frequent, generalized seizures, status epilepticus, and opisthotonos; generalized paralysis or paralysis affecting vital functions; blindness and deafness.

### Prognosis evaluation

We evaluated the prognosis of the patients according to the Glasgow Coma Scale (GCS) and scored each patient. When the patient’s score was greater than or equal to 9 points, the patient’s prognosis was considered good. When the patient’s score was less than or equal to 8 points, the patient’s prognosis was considered poor.

### Image analysis

All images were evaluated by two experienced radiologists in CNS imaging diagnosis, and imaging signs of CT or MRI appearing in patients with hydrogen sulfide poisoning were summarized and analyzed, including generalized brain edema, symmetrical hypodensity/abnormal signals in the bilateral basal ganglia and around the lateral ventricles, subarachnoid or intracerebral hemorrhage, and cerebellar tonsillar herniation; final results were agreed upon by the two radiologists after consultation. The patients were staged according to the gas inhalation time (stage 1: 0–2 weeks; stage 2: 2 weeks to 1 month; stage 3: 1–2 months; stage 4: 2–6 months), and analyses were made on the proportions of the above four signs in patients according to the different grades of clinical symptoms and different time stages, as well as their correlations with clinical grade and patient prognosis.

### Statistical analysis

Statistical analysis was performed using SPSS 16.0 software. Quantitative data are represented as the mean ± standard deviation (SD), and qualitative data are represented as numbers or percentages. Spearman’s correlation analysis was used to analyze correlations of imaging signs of patients with their clinical grading and prognoses, and significance level was *P* < 0.05.

## Results

### General information

According to the central nervous system symptom scores in the Poisoning Severity Score (PSS), 10 clinically minor patients were enrolled in the present study, and their clinical manifestations included drowsiness, vertigo, mild extrapyramidal symptoms, and mild visual or auditory disturbances. There were 17 clinically moderate patients, and their clinical manifestations included unconsciousness with appropriate response to pain, and infrequent, generalized or local seizures. There were 13 clinically severe patients, and their clinical manifestations included deep coma with inappropriate response to pain or unresponsive to pain, respiratory depression with insufficiency, and frequent, generalized seizures.

According to the prognostic evaluation, there were 31 patients with good prognoses and 9 with poor prognoses in this cohort (Table 1).

**Table 1:** Summary of clinical grading, imaging signs, and prognoses of patients

		Imaging sign				Frequency of sign	Patient’s prognosis (GCS)
Clinical grading	Patient (*N* = 40)	Generalized brain edema	Symmetrical hypodense/abnormal intensities in bilateral basal ganglia and around lateral ventricles	Subarachnoid or intracerebral hemorrhage	Cerebellar tonsillar herniation		
Severe (*N* = 13)	1		√			1	Poor (3)
	2		√			1	Poor (7)
	3	√		√		2	Poor (3)
	4	√	√			2	Poor (3)
	5		√			1	Good (15)
	6	√	√	√		3	Poor (3)
	7	√				1	Good (15)
	8					0	Good (15)
	9					0	Good (15)
	10	√		√	√	3	Poor (3)
	11	√		√	√	3	Poor (3)
	12		√			1	Poor (4)
	13		√			1	Poor (5)
Moderate (*N* = 17)	14					0	Good (15)
	15	√				1	Good (15)
	16					0	Good (15)
	17					0	Good (15)
	18					0	Good (15)
	19					0	Good (15)
	20					0	Good (15)
	21					0	Good (15)
	22					0	Good (15)
	23					0	Good (15)
	24					0	Good (15)
	25					0	Good (15)
	26					0	Good (15)
	27					0	Good (15)
	28					0	Good (15)
	29					0	Good (15)
	30					0	Good (15)
Minor (*N* = 10)	31					0	Good (15)
	32					0	Good (15)
	33					0	Good (15)
	34					0	Good (15)
	35					0	Good (15)
	36					0	Good (15)
	37					0	Good (15)
	38					0	Good (15)
	39					0	Good (15)
	40					0	Good (15)

“√” denotes imaging signs appeared in the patient

### Analysis of imaging signs and proportions of patients with different grades of clinical symptoms based on stages according to gas inhalation time

In patients with minor clinical symptoms, CT/MRI of the brain revealed no abnormal findings at the four stages observed.

In patients with moderate clinical symptoms, CT/MRI of the brain revealed imaging findings of brain edema at stage 1 and its absorption at stage 2 in only one patient (7.14%).

**Fig. 1:**
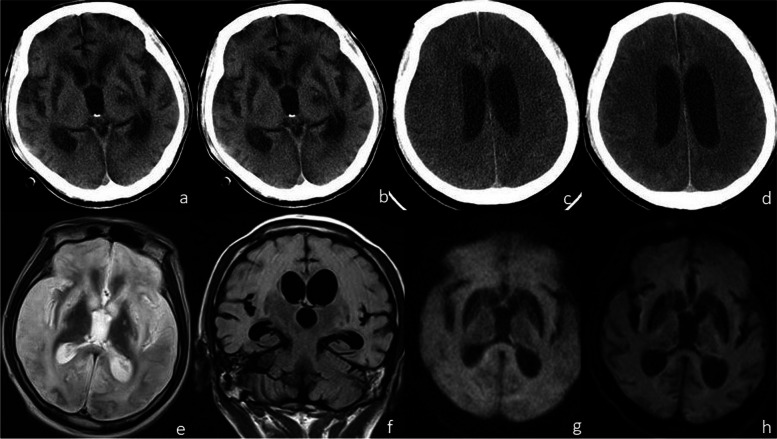
A 41-year-old male septic tank worker who fainted immediately and lost consciousness after H_2_S inhalation was rescued after 50 min but showed lip cyanosis, lock-jaw, and limb tics. He was still in a coma after symptomatic treatment in another hospital. He had a fever after 2 days. On admission, he was in a deep coma, the muscle strength of the four limbs was grade I, and the muscle tension of both arms was enhanced compared with normal. Plain CT scan of the brain revealed symmetrical stripes and patch hypodensities in the bilateral basal ganglia and around the lateral ventricles 45 days after H_2_S inhalation (**a**); CT of the brain was performed 60 days and 4 months after H_2_S inhalation (**b** and **c**), and generalized brain edema appeared and continued to aggravate (CT values were 25 and 17 HU, respectively); CT was performed again after 5.5 months (**d**), on which the severity of brain edema was slightly alleviated, and partial corticomedullary differentiation was slightly clearer than before; on MRI of the brain 4 months after H_2_S inhalation (**e**-**g**), the bilateral basal ganglia were symmetrically hypointense on T1WI and hyperintense on T2WI (**e**), the brain edema showed diffuse hyperintense signals on T2WI and involved the cortex and the white matter, T2WI-Flair (**f**) and DWI (**g**) revealed diffuse hyperintensities, and the basal ganglia lesions were hypointense; MRI of the brain performed after 5.5 months (**h**) revealed a slightly lower degree of hyperintensity on DWI than before. The patient was persistently in an agrypnocoma during follow-up

**Fig. 2:**
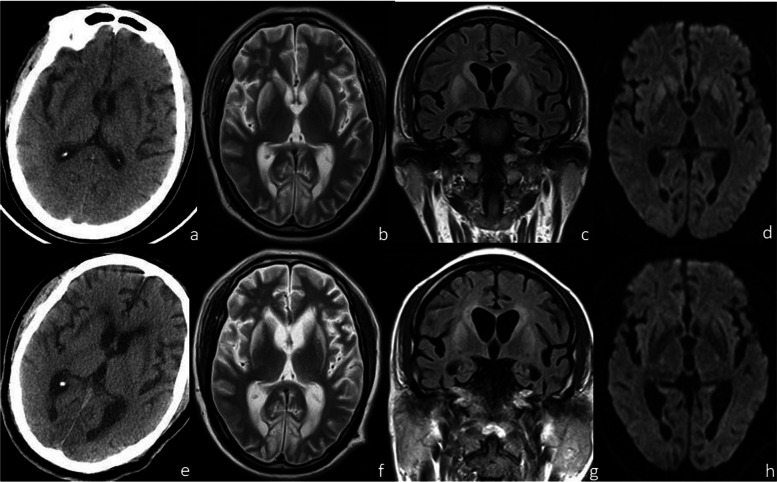
A 49-year-old male who had unconsciousness and of limb tics after H_2_S inhalation in a fish hold was admitted to our hospital after 51 days of treatment in another hospital and was still in a coma and had hypermyotonia in the four limbs on admission. CT of the brain was performed 53 days after the poisoning and revealed symmetrical stripes and patchy hypodensities in the bilateral basal ganglia (**a**); MRI on the same day revealed symmetrical hypointensities on T1WI and hyperintensities on T2WI around the lateral ventricles and in the bilateral basal ganglia (putamen and head of the caudate nucleus) (**b**), and the lesions were slightly hyperintense on T2WI-FLAIR (**c**) and DWI (**d**); CT of the brain 93 days after the poisoning was comparable to the previous CT (**e**), and MRI on the same day revealed that the ranges of lesions on T2WI (**f**) and T2WI-FLAIR (**g**) were also comparable to those on the previous MRI, but reduced hyperintensities were revealed on DWI (**h**); MRI of the brain performed 6 months after poisoning revealed that there were still no significant changes in the lesions. The patient had a poor prognosis and was in a persistent coma

**Fig. 3:**
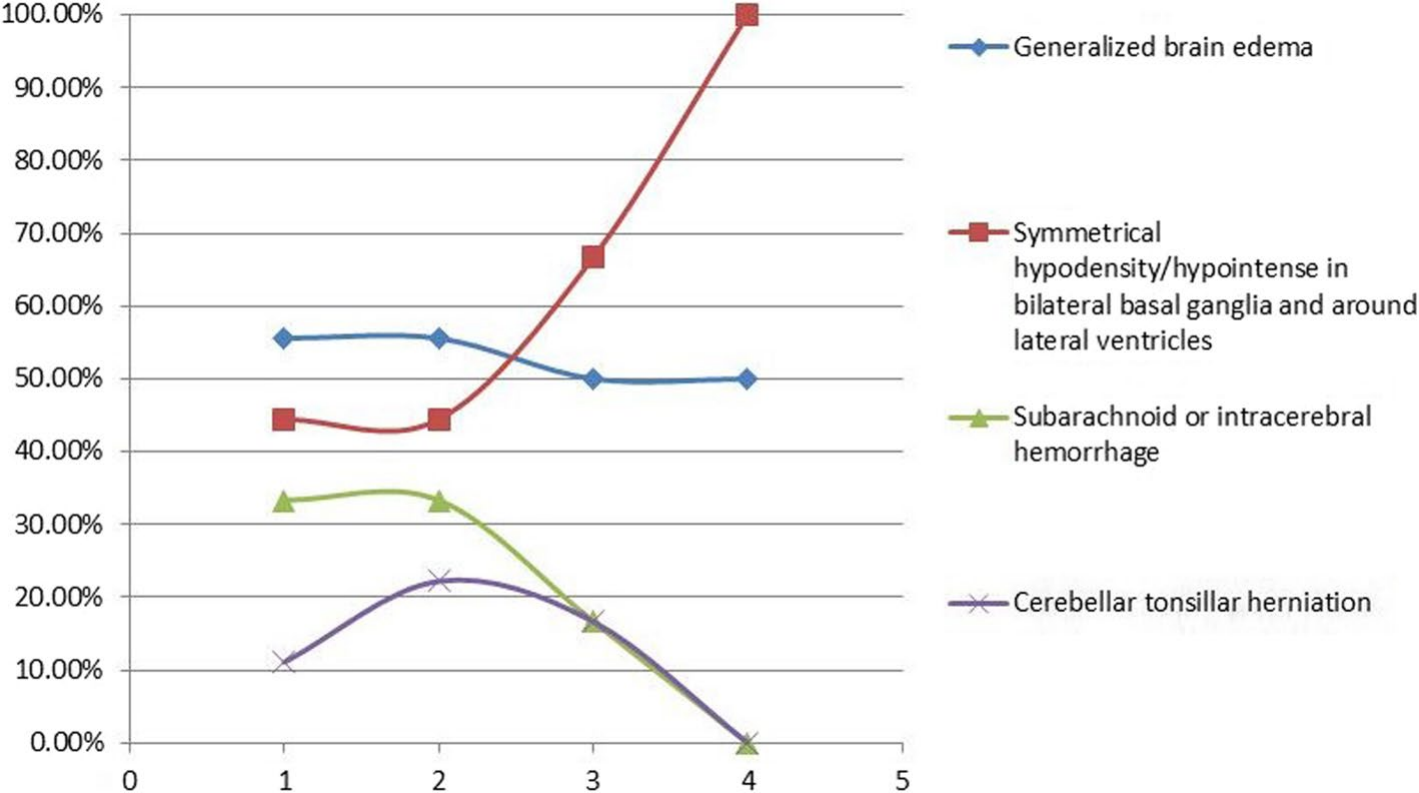
Proportional changes in the findings of severe patients at different time stages. NOTE: The horizontal axis shows the time stages (Stages 1–4)

In patients with severe clinical symptoms, CT/MRI of the brain revealed all four signs (generalized brain edema, symmetrical hypodensity/abnormal signals in bilateral basal ganglia and around lateral ventricles, subarachnoid or intracerebral hemorrhage, and cerebellar tonsillar herniation) at the first three stages (within 2 months); generalized brain edema (Fig. 1) and symmetrical hypodensity/abnormal signals in the bilateral basal ganglia and around the lateral ventricles (Fig. 2) were the main findings; moreover, at the first two stages, the incidence of brain edema was as high as 55.56%, and that of symmetrical hypodensity/abnormal signals in the bilateral basal ganglia and around the lateral ventricles was up to 44.45%, so the incidence of brain edema was slightly higher; at stage 3 and stage 4, the incidence of brain edema decreased slightly (50%), and the main finding was symmetrical hypodensity/abnormal signals in the bilateral basal ganglia and around the lateral ventricles (which rose up to 100% at stage 4); other secondary signs gradually vanished (dropping from a maximum of 33 to 0%) as the follow-up and treatment times increased (Table 2 and Fig. 3).

**Table 2:** Analysis of imaging features based on stages according to different gas inhalation time and clinical grading

Stage 1 (0–2 weeks)			
Imaging sign	Clinical grading: Minor (*N* = 10)	Clinical grading: Moderate (*N* = 14)	Clinical grading: Severe (*N* = 9)
Generalized brain edema	0	1 (7.14%)	5 (55.56%)
Symmetrical hypodense/abnormal intensities in bilateral basal ganglia and around lateral ventricles	0	0	4 (44.45%)
Subarachnoid or intracerebral hemorrhage	0	0	3 (33.33%)
Cerebellar tonsillar herniation	0	0	1 (11.11%)
stage 2 (2 weeks to 1 month)			
Imaging sign	Clinical grading: Minor (*N* = 3)	Clinical grading: Moderate (*N* = 10)	Clinical grading: Severe (*N* = 9)
Generalized brain edema	0	0	5 (55.56%)
Symmetrical hypodense/abnormal intensities in bilateral basal ganglia and around lateral ventricles	0	0	4 (44.45%)
Subarachnoid or intracerebral hemorrhage	0	0	3 (33.33%)
Cerebellar tonsillar herniation	0	0	2 (22.22%)
stage 3 (1–2 months)			
Imaging sign	Clinical grading: Minor (*N* = 0)	Clinical grading: Moderate (*N* = 6)	Clinical grading: Severe (*N* = 6)
Generalized brain edema	0	0	3 (50.00%)
Symmetrical hypodense/abnormal intensities in bilateral basal ganglia and around lateral ventricles	0	0	4 (66.67%)
Subarachnoid or intracerebral hemorrhage	0		1 (16.67%)
Cerebellar tonsillar herniation	0	0	1 (16.67%)
stage 3 (2–6 months)			
Imaging sign	Clinical grading: Minor (*N* = 0)	Clinical grading: Moderate (*N* = 4)	Clinical grading: Severe (*N* = 2)
Generalized brain edema	0	0	1 (50%)
Symmetrical hypodense/abnormal intensities in bilateral basal ganglia and around lateral ventricles	0	0	2 (100%)
Subarachnoid or intracerebral hemorrhage	0	0	0
Cerebellar tonsillar herniation	0	0	0

### Correlations of patient imaging signs with clinical grade (minor, moderate, and severe) and prognosis

Minor, moderate, and severe clinical grading patients were divided into two groups; the minor and moderate clinical grade group, with 27 patients, and the severe clinical grade group, with 13 patients. An analysis was performed on the correlations between the four imaging features (generalized brain edema, symmetrical hypodensity/abnormal signals in bilateral basal ganglia and around lateral ventricles, subarachnoid or intracerebral hemorrhage, and cerebellar tonsillar herniation) and clinical grading results. The results showed a significant correlation between imaging signs and severe clinical grading (*P* = 0.000, R = 0.828).

The prognosis results of the patients were divided into two groups; the good prognosis group, with 31 patients, the poor prognosis group, with 9 patients. An analysis was performed on the correlations between the four imaging features and the prognosis results of the patients, and the results showed a significant correlation between imaging signs and a poor prognosis (*P* = 0.000, R = 0.858).

## Discussion

Hydrogen sulfide, an irritant toxic gas, can cause neural inhibition and respiratory depression to rapidly leading to loss of consciousness and thus “lightning-like” death at higher concentrations [9, 10]; its concentration and exposure time determine the degree of injury, and the tissue most sensitive to its toxicity is brain tissue, which needs more oxygen [11, 12]; however, the most commonly affected sites in the brain are the nuclei of the bilateral basal ganglia; because these nuclei require a large quantity of oxygen, imaging findings show symmetrical hypodensities/intensities in the bilateral basal ganglia and around the lateral ventricles; lesions are distributed in the bilateral frontoparietal white matter, centrum ovale, periphery of the lateral ventricles, and nuclei of the basal ganglia; MRI reveals symmetrical hypointensities on T1WI and hyperintensities on T2WI; DWI and T2-FLAIR images show symmetrical hypointensities, and the cortex is extensively involved in some patients; T2-FLAIR images show hypointensities, and involvement of basal ganglia nuclei is dominant; moreover, MRI reveals lesions earlier than CT and with a clearer range. In general, a higher concentration means more significant central nervous depressant activity. The actions of inhaled H_2_S on cytochrome oxidase in the respiratory chain and disulfide bonds hinder the cellular redox process, causing cellular “internal asphyxia” in the tissue, resulting in hypoxia and high oxygen consumption by brain tissue. Therefore, the brain tissue is the most sensitive to hypoxia, and brain dysfunction will occur at the early stage of poisoning [13, 14]. Hypoxia not only causes a hindered bio-oxidation process, energy generation disorder, intracellular water-sodium retention, acidosis, and increased intracellular H^+^ but also activates the Na-Ca exchange mechanism by enhancing H^+^-Na^+^ exchange, causes intracellular calcium overload, induces xanthine dehydrogenase to transform into xanthine oxidase, enables the body to produce a large amount of superoxide anions in the process of metabolizing purine compounds into uric acid, and thus induces lipid peroxidation damage. This may be the most important molecular mechanism underlying hypoxic damage [15, 16]. In addition, acute poisoning induced by H_2_S is accompanied by brain edema, and its pathogenesis involves energy metabolism disorders of the brain, abnormal distribution of ions inside and outside brain cells, dysfunction of the blood-brain barrier, and adverse effects of free arachidonic acid and free radicals [17–19]. Cytotoxic brain edema occurs first, i.e., intracellular edema. CT findings reveal diffuse cerebral parenchymal hypodensity, ill-defined corticomedullary differentiation, and shallow cerebral sulci, which are mainly distributed in the basal ganglia, around the lateral ventricle, and in the centrum ovale; the cortex and the subcortical white matter may also be hypodense; MRI reveals hypointensities on T1WI and hyperintensities on T2WI, ill-defined boundaries, and hyperintensities on DWI. Vasogenic brain edema, i.e., extracellular edema, occurs subsequently over time, during which hyperintensities on DWI are attenuated to iso- or hypointensities. Therefore, the effect of DWI on MRI should not be neglected. Because poisoning per se causes vascular injury, patients may have subarachnoid or brainstem hemorrhage. All mechanisms are unclear. Cerebral herniation is a secondary change that is mostly caused by intracranial hypertension and is not specific. The appearance of the latter two imaging signs is suggestive of a severe condition and the prognosis tends to be poor.

Among the patients studied, in those with minor clinical symptoms, CT/MRI of the brain revealed no abnormal findings at any of the four stages observed. In patients with moderate clinical symptoms, CT/MRI of the brain revealed imaging findings of brain edema at stage 1 and its absorption at stage 2 in only one patient (7.14%). In patients with severe clinical symptoms, CT/MRI of the brain revealed all four imaging signs at the first three stages; generalized brain edema and symmetrical hypodensity/abnormal signals in the bilateral basal ganglia and around the lateral ventricles were the main findings; moreover, at the first two stages, the incidence of brain edema was up to 55.56%, and that of symmetrical hypodensity/abnormal signals in the bilateral basal ganglia and around the lateral ventricles was up to 44.45%, so the incidence of brain edema was slightly higher, unsurprising as it is caused by intra- and extracellular edema at the acute stage; at stage 3 and stage 4, the main finding was symmetrical hypodensity/abnormal signals in the bilateral basal ganglia and around lateral ventricles (which rose to 100% at stage 4), likely due to the high dose of H_2_S, severe hypoxia, long hypoxic time of brain tissue, and irreversible organic damage in brain cells. Therefore, the incidence of symmetrical hypodensity/abnormal signals in the bilateral basal ganglia and around the lateral ventricles did not drop but instead rose, while that of brain edema (50%) dropped slightly, indicating that the brain edema was absorbed insignificantly as the duration of poisoning increased. Moreover, accompanying subarachnoid or intracerebral hemorrhage and cerebellar tonsillar herniation may also aggravate the disease, which may be one of the causes of a poor prognosis. Statistical analysis demonstrated that these four imaging signs were significantly correlated with clinical severity, suggesting that positive treatment should be performed clinically.

Among the 40 patients, there were a total of 10 with minor clinical grade, and their findings revealed no abnormalities with good prognoses. There were 17 patients with moderate clinical grade; CT/MRI of the brain revealed imaging findings of brain edema at stage 1 and its absorption at stage 2 in only one patient, and these 17 patients also had good prognoses. Among 13 patients with severe clinical grade, nine had poor prognoses, and four had good prognoses. Among the four patients with good prognoses, two had no abnormal findings. One revealed no abnormalities on CT in another hospital on the day of inhalation, MRI at stage 1 revealed symmetrical hypointensities on T1WI and hyperintensities on T2WI in the bilateral basal ganglia (globus pallidus) and a slight hyperintensities on DWI; later CT and MRI were not reviewed. In the other patient, CT at stage 1 revealed symmetrical hypodensities in the bilateral cerebella and brachium pontis with a CT value of 20 HU, shallow adjacent cerebral sulci, and slight hypodensities in the right basal ganglia and the white matter around the lateral ventricles, likely due to the localized brain edema. CT was reviewed 1 week later, and the above findings were absorbed, suggesting that low doses and concentration of inhaled hydrogen sulfide might not cause significant brain injury, because the prognosis was also good. Statistical analysis demonstrated that these four imaging signs were significantly correlated with a poor prognoses. Patients who had these four imaging signs for extended periods of time had poor prognoses and should be of high clinical concern.

There are some shortcomings in the present study. First, the present study was retrospective in nature. Second, the patients in the cohort were not followed up regularly, and some patients were lost to follow-up at the late stage, possibly because the patients were too severe to receive examinations or had died. Third, for some patients, CT and MRI of the brain were not performed simultaneously, so only CT or MR images were available; the MRI scan clearly revealed abnormal signals in the bilateral basal ganglia and the extent of brain edema.

## Conclusions

In conclusion, the CT/MRI imaging features of the brain in patients with hydrogen sulfide poisoning mainly consist of four signs that have a high probability of being found in patients with severe clinical grade; generalized brain edema and symmetrical hypodensity/abnormal signals in the bilateral basal ganglia and around the lateral ventricles are the main findings and were shown to persist. The imaging signs are correlated with the severe clinical grade and a poor prognosis and can thus partly assist in clinically and effectively evaluating clinical symptom grade, performing early diagnosis, and administering timely treatment.

## Data Availability

Anonymized data from the present study will be shared on reasonable request from any qualified researcher for well-defined research questions. Please contact the corresponding author.
